# CROSS-SECTIONAL AND LONGITUDINAL RELATIONSHIPS BETWEEN REST-ACTIVITY RHYTHMS AND INFLAMMATION IN OLDER MEN

**DOI:** 10.1093/geroni/igz038.1497

**Published:** 2019-11-08

**Authors:** Xiao Qian, Qian Xiao, Daniel S Evans, Susan Redline, Nancy Lane, Sonia Ancoli-Israel, Katie S Stone

**Affiliations:** 1 University of Iowa, Iowa City, Iowa, United States; 2 California Pacific Medical Center Research Institute, San Francisco, California, United States; 3 Brigham and Women’s Hospital, Boston, Massachusetts, United States; 4 UC Davis, Davis, California, United States; 5 University of California San Diego, La Jolla, California, United States

## Abstract

Sleep disturbances and physical inactivity have been associated with chronic inflammation, an important risk factor for cognitive decline in the aging population. However most previous studies focused on the cross-sectional relationships between sleep and physical activity and inflammation. In the Outcomes of Sleep Disorders in Older Men (MrOS Sleep) study, we studied both the cross-sectional and prospective associations between characteristics of 24-hour rest-activity rhythms measured by actigraphy and inflammation index measured by multiple circulating markers. In cross-sectional analysis, a lower amplitude is associated with elevated inflammation (Odds ratio Q4 vs Q1 (95% Confidence interval): 1.65 (1.22, 2.24)). In prospective analysis, an earlier acrophase (<12:30) is associated with a two-fold increase in the risk of developing elevated inflammation over four years of follow up (2.08 (1.02, 4.23)). No individual inflammatory markers are associated with rest-activity rhythms. Our findings suggest that rest-activity rhythm characteristics predicts elevated inflammation.

